# The impact of teacher care on teacher-student relationship: evidence from cross-sectional and longitudinal data

**DOI:** 10.3389/fpsyg.2025.1551081

**Published:** 2025-01-28

**Authors:** Zhen Zhang, Yuxin Wang, Wenqing Deng, Xueling Ma, Chunhui Qi

**Affiliations:** ^1^Faculty of Education, Henan Normal University, Xinxiang, China; ^2^Faculty of Education, Henan University, Kaifeng, China

**Keywords:** teacher care, teacher-student relationship, teacher gender, cross-sectional, longitudinal

## Abstract

Based on a sample of middle school students from the China Education Panel Survey (CEPS) database, this study explores the relationship between parental perceived teacher care and teacher-student relationship and the role of teacher gender in it through cross-sectional and longitudinal studies. Study 1 found that there was a significant positive correlation between teacher gender, parental perceived teacher care and teacher-student relationship. Analysis of simple moderation effects suggests that teacher gender plays a moderating role in the relationship between parental perceived teacher care and teacher-student relationships. Subsequent simple slope analysis indicates that the influence of female teacher care on teacher-student relationship is notably stronger than that of male teacher care. Study 2 Descriptive statistical results indicated that during the seventh and eighth grades of Chinese middle school students, the level of teacher care showed a slight decrease, and teacher-student relationship showed a decreasing trend. Correlational results demonstrated a significant positive correlation between parental perceived teacher care and teacher-student relationship at both time points. Further analysis using cross-lagged models revealed that teacher care significantly positively predicted subsequent teacher-student relationship, and teacher-student relationship significantly positively predicted subsequent teacher care. This bidirectional predictive relationship did not have the moderation of teacher gender. Based on these findings, educators should strengthen the communication and cooperation between parents and teachers, and create a benign educational atmosphere for student interaction in school and family.

## 1 Introduction

The teacher-student relationship (TSR) is a type of interpersonal relationship that gradually develops between teachers and students through communication and interaction, primarily manifesting in emotional, cognitive, and behavioral communication (Wang X. et al., 2024). As one of the most critical and fundamental interpersonal relationships in schools, a good TSR is of special significance for the growth and development of adolescents (Ma et al., 2022). Previous studies have shown that teacher characteristics are an important indicator of TSR (Doyle et al., 2022). This does not mean that all of the teacher’s behavioral characteristics can have an impact on TSR, but that some of the teacher’s behaviors that are closely related to the student can have an effect on the establishment of a good TSR. Research on the impact of teacher factors on TSR has shifted from studying teachers’ negative behaviors (e.g., punishment for failure, not allowing students to have independent opinions, etc.) to studying the positive predictive effect of teachers’ positive behaviors (e.g., teacher support, teacher self-efficacy) on TSR (Hajovsky et al., 2020; Sun and Shi, 2022). However, in addition to the above-mentioned positive factors such as teacher support and teacher self-efficacy, which can positively predict TSR, the importance of teacher care in TSR cannot be ignored, and few researchers have explored it.

Teacher care refers to the emotional support, attention, and empathy extended by teachers to their students (Zhou, 2023). Although there are few studies on teacher care and TSR, attachment theory can explain the relationship between them to some extent. This theory suggests that children’s interactions with their caregivers form an internal working model that affects their future intimate relationships. When caregivers are able to meet the needs of children in a timely and effective manner, giving them care and support, children and caregivers are able to establish secure attachment relationships (Ali et al., 2021). Teachers are important attachment figures for students, especially in adolescence (Ansari et al., 2020). As students get older, adolescents may rely less on parent–child relationships and more on teacher-student relationships. TSR can be regarded as an extension of the parent–child relationship (Yang et al., 2024). It can be seen that in the context of education, teachers, as the people with the closest relationship with students, should have a significant correlation with the relationship between teachers and students. This assumption is supported by empirical studies, such as the finding that teacher expectations are a positive predictor of TSR (Trang and Hansen, 2021). In general, teacher expectations need to be expressed through the words and actions of teachers, especially through the words and deeds of teachers who care for their students. That is to say, when teachers give students caring behavior, students and teachers form bidirectional teacher-student interaction in continuous communication, which in turn increases the intimacy between students and teachers, reduces conflicts, and establishes a high-quality TSR.

Noddings (2001) proposes the idea of care theory, which argues that care in TSR implies a relationship, a connection or contact between two people where one party gives care and the other receives it. This relationality suggests that teacher care and TSR are interdependent (Derakhshan et al., 2022). Current research indicates that TSR also has a positive impact on teachers, which can enhance their motivation, effort, engagement, well-being, and confidence, thus prompting teachers to pay more attention to students’ needs, interests and feelings (van der Lans et al., 2020). Although we have some understanding of the relationship between teacher care and TSR, there is still insufficient empirical evidence of the bidirectional relationship between these two variables in the context of Chinese education. For middle school students, particularly those in the crucial stage of grade 7, a profound comprehension of the significance of TSR and teacher care is especially vital, as adolescents in this age group are in a critical period of mental health development, academic adaptation, and personality development (Cao and Liu, 2023). At this stage, they are not only facing academic pressure and social challenges, but they are also exploring the formation of self-identity and values. A good TSR can provide students with emotional support and a sense of security, helping them better cope with these challenges (Saxer et al., 2024). Meanwhile, teacher care can boost students’ self-confidence and motivation for learning, thereby facilitating their academic performance and mental health (Lavy and Naama-Ghanayim, 2020). Therefore, building a good TSR and providing effective teacher care is not only crucial for students’ academic success, but also has a profound impact on their overall development and future social adaptability.

When examining the average level of the quality of TSR, studies consistently show that teachers perceive their relationships with female students to be more positive than those with male students (Walker and Graham, 2021; Brinkworth et al., 2018). Compared to student gender, the role of teacher gender in teacher-student relationships has been far less examined (Roorda and Jak, 2024). According to the socialization theory of gender roles, men and women have different roles and responsibilities in the socialization process, and gender differences will be manifested in many aspects (Andreeva and Zappa, 2023). Research has shown that male and female teachers may differ in their attitudes and behaviors toward students. For example, female teachers may give more attention to students’ emotional needs, while male teachers may devote more attention to students’ academic performance (Denessen et al., 2022). Although care is considered an educational responsibility that should be shared by both male and female teachers, males seem to have the prerogative to decide whether or not to engage in caring behaviors (Lahelma et al., 2014). In comparison, female teachers often shoulder heavier responsibilities, either voluntarily or involuntarily, which include close personal emotional or verbal interactions (Lu, 2018). This is because females traditionally place a greater emphasis on establishing emotionally intimate relationships (Bonhag and Froese, 2022; Löffler and Greitemeyer, 2023). Consequently, female teachers may potentially have more advantageous relationships with students compared to male teachers. This is supported by some primary school studies, with pupils suggesting that they are more closely associated with female teachers than with male teachers (Charki et al., 2022; Zee et al., 2020). However, the current evidence is limited to primary education, and it is unclear whether teacher gender plays a role in the TSR in secondary education. Therefore, whether the difference in teacher gender in middle schools can moderate the bidirectional relationship between teacher care and TSR is also a question worthy of exploration.

However, the majority of existing studies have measured teacher care from the perspectives of teachers and students (Derakhshan et al., 2022; Li and Chu, 2023). There is a lack of research measuring teacher care from the subjective perception of parents as a third party. When students evaluate teacher care or TSR, maintaining their social desirability may influence their responses. This tendency may lead to certain biases in self-assessment. In contrast, parents as third-party evaluators can avoid such biases to a certain extent. Moreover, as a trend in educational development, the collaboration between home and school has gradually become a hot topic in educational research (Krüger and Thamin, 2021; Santamaría Graff, 2021; Lu and Yu, 2024), with home-school cooperation playing a positive and promotive role in the education of adolescents. The student care initiated by teachers in conjunction with parents is an important direction. For instance, when teachers communicate with parents about children’s academic performance, psychological state, and social interactions, it sends a signal of care to the parents regarding their own children. Research has shown that when teachers have a close relationship with children, parents are also more likely to observe the interactions between teachers and students and obtain more positive feedback (Westerberg et al., 2020). Therefore, parents’ perceptions also help to reveal the adaptation of students between home and school, and further understand the impact of teacher care on students’ development. At the same time, in the CEPS data used in this study, only reports provided by parents are available for measuring teacher care and TSR, without involving self-assessments from students and teachers.

Therefore, based on the availability of existing data and the rationality and significance of parents as third-party evaluators, this study aims to systematically explore the relationship between parental perceived teacher care and TSR through 2 studies, and to reveal the key role of teacher gender and teacher care in the development of TSR in middle school in China. Study 1 aims to validate the mechanism by which parental perceived teacher care affects TSR among middle school students in China, based on cross-sectional data from the CEPS. It also further explores the moderating role of teacher gender in this process. Study 2 continues with a one-year longitudinal tracking study of middle school students, conducting two surveys over the year, and employs a cross-lagged model to clarify the bidirectional predictive relationships between parental perceived teacher care and TSR among Chinese middle school students, as well as to determine if teacher gender plays a moderating role in it. Based on the literature and research objectives, we propose the following research hypotheses:

*Hypothesis 1*: There is a positive correlation between teacher care and TSR.

*Hypothesis 2*: There is a bidirectional relationship between teacher care and TSR among Chinese middle school students.

*Hypothesis 3*: Teacher gender plays a moderating role between teacher care and TSR.

## 2 Method

### 2.1 Participants

The dataset utilized in this study is derived from the CEPS, a nationally representative longitudinal survey that has garnered significant acclaim. CEPS was meticulously designed by the National Survey Research Center (NSRC) at Renmin University of China. The survey, which is ongoing, has thus far released data from two distinct waves, corresponding to the academic years of 2013–2014 and 2014–2015. With the 2013–2014 academic year serving as the reference point, CEPS initiated the survey with two concurrent cohorts: middle school students in the seventh grade and ninth grade. The sampling methodology incorporated a multistage probability proportional to size (PPS) approach, coupled with whole-class sampling. Stratification variables included average educational attainment and the proportion of the floating population. Subsequent to the initial survey, a follow-up was conducted during the 2014–2015 academic year, focusing exclusively on students who were in the seventh grade at the outset of the study. The participants in this dataset were all anonymous and voluntary, identified only through coding. The survey was designed with separate questionnaires for different target groups, including students, parents, head teachers, subject teachers, and school leaders. Except for parents, all other target groups were required to fill out the questionnaires collectively on site. There are two ways for parents to fill out the questionnaire: (a) students take the questionnaire home, parents fill it out individually, and then students bring it back to school; (b) head teachers organize parents to fill out the questionnaire collectively during parent meetings and collect them on the spot. The survey was conducted on a school-based basis, with 112 schools and 438 classes randomly selected from the chosen county-level units for investigation. All students in the selected classes were included in the sample, and the baseline survey involved approximately 20,000 students.

The data sources are relatively old, and the outbreak of the COVID-19 pandemic may have affected parental perceived teacher care and TSR. Many parents have indicated that in virtual teaching environments, teachers are unable to show sufficient emotional care as they can in face-to-face teaching, and the relationships between teachers and students have also been affected (El-Metaal and Sanchez, 2024). However, the purpose of this study is to explore the basic association between parental perceived teacher care and TSR. Therefore, the focus of our study is on the educational environment before the pandemic, rather than the specific impact of the pandemic on these variables. Considering that the survey included the basic demographic characteristics of students, teacher gender, teacher care and TSR reported by parents, it was able to provide good data support for the analysis of research questions in this study. Therefore, this study was analyzed based on CEPS.

Participant selection method for this study: First, since the study targets Chinese middle school students, we included demographic characteristics such as gender, date of birth, and class level of the students as control variables in the research. Second, we selected the three variables to be explored in this study: teacher care, teacher-student relationship, and teacher gender. We chose the measurement items for teacher care and teacher-student relationship from the parent questionnaire. Since Study 2 further tracked the changes of the same group of samples, the sample selection was consistent with Study 1, with the only difference being the addition of time dimension measurements.

In Study 1, we selected the baseline data from 2013–2014, which included seventh and ninth graders, for preliminary exploration. There were a total of 19,487 samples. After excluding samples with key missing values, the final valid sample size was 17,666, consisting of 8,900 male students and 8,766 female students, with an average age of 13.50. Among the teachers, there were 6,476 male teachers and 11,190 female teachers.

In Study 2, we selected students who participated in both waves of the survey, which are grade seven at baseline and grade eight at the follow-up survey. We also excluded samples with missing variables. The final sample consisted of 5,939 students. There were 2,724 boys and 2,669 girls, and the average age of the students was 13.48. There were 1,681 male teachers and 3,712 female teachers. The study was approved by the Ethics Committee of the Faculty of Education, Henan Normal University. For the research data, please refer to the Supplementary materials.

### 2.2 Measures

#### 2.2.1 Teacher care

To measure the levels of teacher care, we employed two items from the CEPS parent questionnaire: “The teacher’s sense of responsibility toward the child,” with response options on a 5-point Likert scale ranging from “1 = Not at all responsible” to “5 = Very responsible”; and “The teacher’s patience with the child,” with responses ranging from “1 = Not at all patient” to “5 = Very patient.” The average score across these two questions was calculated, with higher values indicating a higher level of teacher care. The reliability coefficients *α* for teacher care in the first and second years were 0.87 and 0.86, respectively, indicating a high level of measurement reliability.

#### 2.2.2 Teacher-student relationship

To measure the levels of teacher-student relationship, we employed two items from the CEPS parent questionnaire: “Does the child like their homeroom teacher?” and “Does the child like other teachers?” Each question offered four response options: “1 = Not at all,” “2 = Not very much,” “3 = Fairly,” and “4 = Very much.” The average score of these two items was calculated, with higher scores indicating a better TSR. The reliability coefficients α for TSR in the first and second years were 0.78 and 0.74, respectively, indicating a high level of measurement reliability.

### 2.3 Data analysis

This study used SPSS 26.0 and Mplus 8.1 software for data analysis. The dataset used was sourced from the CEPS database, which provided the secondary data for our analysis. We first conducted a descriptive analysis of teacher care and TSR among Chinese middle school students to gain a basic understanding of these variables. Building upon this foundation, we conducted a correlation analysis to examine the associations between teacher care and TSR. Finally, in line with the research objectives and hypotheses, in Study 1, we utilized Model 1 from the SPSS macro program PROCESS 4.1 to further investigate the moderating effect of teacher gender on the relationship between teacher care and TSR; In Study 2, we used a cross-lagged model to analyze the bidirectional predictive relationships between teacher care and TSR, as well as to examine whether there are gender differences in this bidirectional relationship among teachers.

### 2.4 Cross-lagged model

The cross-lagged model, a longitudinal research model, is utilized to scrutinize the existence and direction of relationships between variables. Within this model’s framework, if X and Y represent two variables, and T1 and T2 represent two points in time (T1 is earlier than T2), the cross-lagged model essentially compares the relationship between X at time T1 and Y at time T2 and the relationship between Y at time T1 and X at time T2, thereby elucidating clarify how X and Y interact with each other. In the current study, we concentrate on the variables of teacher care and TSR. Consequently, we employ the cross-lagged model to explore the predictive effects of teacher care on TSR and to delineate the bidirectional relationship between these variables among Chinese middle school students.

## 3 Study 1 results

### 3.1 Descriptive statistics and correlation analysis

Descriptive statistics and correlation analysis were performed on teacher gender, teacher care, and TSR, and the results are shown in Table 1. There is a significant positive relationship between teacher gender, teacher care and TSR. Hypothesis 1 was also supported.

**Table 1:** Descriptive statistics and correlation analysis.

Variable	*M*	*SD*	1	2	3
Teacher gender	0.63	0.48	1		
Teacher care	4.26	0.74	0.14^***^	1	
TSR	3.32	0.59	0.13^***^	0.57^***^	1

^*^p < 0.05, ^**^p < 0.01, ^***^p < 0.001.

### 3.2 Simple moderation effect analysis

First, all variables were standardized. Then, based on the results of the correlation analysis, the data met the statistical requirements for further moderation effect testing. This study performed data analysis using version PROCESS 4.1, which is capable of handling complex statistical analysis tasks, such as moderation effects. Moreover, it offers a variety of model options, allowing researchers to select the appropriate model based on the specific needs of their study (Zhang and Qi, 2024; Zhao et al., 2024). Therefore, using the bias-corrected percentile Bootstrap method with 5,000 resamples, we conducted moderation effect analysis with Model 1 from the SPSS macro program PROCESS 4.1, as detailed in Table 2. The analysis revealed that, after controlling for students’ gender, age, and grade level, teacher care positively predicted TSR (*β* = 0.55, *p* < 0.001), teacher gender significantly predicted TSR (*β* = 0.04, *p* < 0.001), and the interaction term between teacher gender and teacher care significantly predicted TSR (*β* = 0.02, *p* < 0.001). Hypothesis 1 and 3 were also supported.

**Table 2:** Simple moderation effect analysis.

Variable		Fitting index			Standardized coefficients		
Result variable	Prediction variable	*R*	*R^2^*	*F*	*β*	*t*	95%CI
TSR	Teacher care	0.58	0.33	1461.97	0.55^***^	86.99	[0.54,0.56]
	Teacher gender				0.04^***^	6.89	[0.03,0.06]
	Teacher care × teacher gender				0.02^***^	3.70	[0.01,0.03]

^*^p < 0.05, ^**^p < 0.01, ^***^p < 0.001.

**Figure 1:**
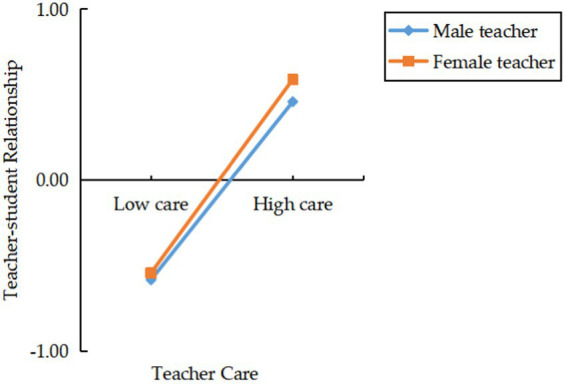
Moderation effect of teacher gender on teacher care and TSR.

Further, simple slope analysis was utilized to elucidate the nature of the interaction effect between teacher gender and teacher care, with a simple slope plot provided for further explanation (see Figure 1). The results indicated that the predictive effect of teacher care on TSR for female teachers (*β* = 0.56, *t* = 69.84, *p* < 0.001) was significantly greater than that for male teachers (*β* = 0.52, *t* = 52.67, *p* < 0.001), suggesting that teacher care exhibited by female teachers has a greater impact on student-teacher relationships.

## 4 Study 2 results

### 4.1 Descriptive statistics of teacher care and TSR

Table 3 presents descriptive statistics, including the mean scores, standard deviations and other indicators, for teacher care and TSR among Chinese middle school students. Paired samples *t*-tests revealed significant differences in teacher care and TSR between the pre-test and post-test assessments. The mean score for teacher care at T2 was significantly lower than at T1 (*p* < 0.001), and similarly, the mean score for TSR at T2 was significantly lower than at T1 (*p* < 0.001). For detailed results, see Table 3. Furthermore, compared to T1, there was a significant decline in both the levels of teacher care and TSR at T2 (*t* = 5.15, *p* < 0.001; *t* = 21.98, *p* < 0.001).

**Table 3:** Descriptive statistics of teacher care and TSR.

Year	Variables	*N*	Mean	Standard deviation	Min	Max	Skewness	Kurtosis
Year1	Teacher care	5,393	4.39	0.68	1	5	−1.079	1.149
	TSR	5,393	3.46	0.54	1	4	−0.695	0.457
Year2	Teacher care	5,393	4.34	0.74	1	5	−1.207	1.690
	TSR	5,393	3.27	0.58	1	4	−0.494	0.401

### 4.2 Correlation analysis of teacher care and TSR

Table 4 shows the correlation coefficients between teacher care and TSR among Chinese middle school students. According to Cohen’s guidelines, correlation coefficients of 0.1, 0.3 and 0.5 are considered small, medium and large effect sizes, respectively (Jacob, 1992). In the first year, the effect size of the relationship between teacher care and TSR was relatively large (*r* = 0.54). In the second year, a similar trend was observed, with the effect size of the relationship being 0.56. There was a significant positive correlation between teacher care (*r* = 0.13) and TSR (*r* = 0.12) at both time points. In summary, it is evident that the positive correlation between teacher care and TSR, remain stable among Chinese middle school students in grades seven and eight. Moreover, these two variables are significantly correlated across different years (*p* < 0.001). Hypothesis 1 was also supported.

**Table 4:** Correlation analysis of teacher care and TSR.

Year	Variables	1	2	3	4
Year1	1. Teacher care	1			
	2. TSR	0.54^***^	1		
Year2	3. Teacher care	0.13^***^	0.13^***^	1	
	4. TSR	0.12^***^	0.12^***^	0.56^***^	1

^*^p < 0.05, ^**^p < 0.01, ^***^p < 0.001.

### 4.3 Cross-lagged model of teacher care and TSR

**Figure 2:**
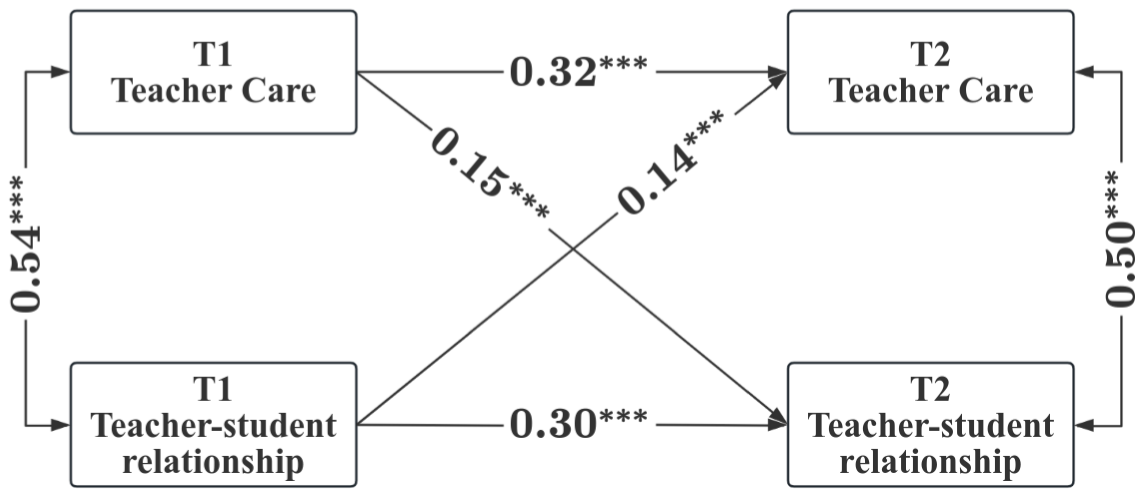
Bidirectional relationships between teacher care and TSR. We use “^*^” to indicate a statistically significant relationship (*p* < 0.05) between variables.

Building on correlation analysis, we utilized Mplus 8.3 software for the analysis, constructing a cross-lagged model to investigate the bidirectional relationship between teacher care and TSR. Mplus version 8.3 is capable of handling large-scale datasets and supports various data types, including longitudinal data. Additionally, it provides a range of model fit indices and diagnostic tools, such as chi-square values, CFI, and RMSEA, which assist in evaluating model fit and identifying potential issues (Koziol, 2023). This ensures the scientificity and credibility of the research results. Given that this model is saturated, with all parameters to be estimated matching the elements in the covariance matrix, resulting in zero degrees of freedom, we did not estimate fit indices and focused solely on the path coefficients (Steeger and Gondoli, 2013). The results of the cross-lagged model are depicted in Figure 2. The standardized autoregressive coefficients revealed significant autoregressive path coefficients from T1 to T2 for both teacher care (*β* = 0.32, *p* < 0.001) and TSR (*β* = 0.32, *p* < 0.001), indicating a certain degree of stability for teacher care and TSR over a one-year period. The standardized cross-lagged coefficients demonstrated a bidirectional predictive relationship between teacher care and TSR, with T1 teacher care significantly and positively predicting T2 TSR (*β* = 0.15, *p* < 0.001), and T1 TSR significantly and positively predicting T2 teacher care (*β* = 0.14, *p* < 0.001). Hypothesis 2 was also confirmed.

### 4.4 Cross-lagged model of teacher gender difference test

To further investigate potential gender differences in the cross-lagged model, we incorporated the moderating variable of teacher gender by creating interaction terms. This was achieved by multiplying the moderating variable of teacher gender with the cross-lagged paths, resulting in two interaction terms: the effect of the product of T1 teacher care and teacher gender on T2 TSR, and the effect of the product of T1 TSR and teacher gender on T2 teacher care. The results indicated that neither of the interaction term path coefficients was significant (*p* = 0.92, *p* = 0.83), suggesting that teacher gender does not moderate the cross-lagged paths within the model. Consequently, Hypothesis 3 was not substantiated.

## 5 Discussion

Study 1, based on the data from the CEPS (2013–2014), empirically explores the impact of teacher care on TSR and its underlying mechanisms. Correlation analysis results indicate a significant positive correlation between teacher care and TSR. Further moderation effect analysis reveals that teacher care can directly predict TSR, and teacher gender plays a moderating role between teacher care and TSR. This is consistent with the study by Oppenhaim-Shachar and Berent (2023), which suggests that when parents perceive that teachers care about their children, they are more likely to establish trust in teachers. This trust forms the foundation of a good TSR. After sensing the teachers’ concern, parents are willing to believe that their children’s teachers genuinely want the children to succeed. Therefore, they are willing to follow the teachers’ guidance and suggestions, communicate and interact with teachers, thereby giving a positive evaluation of the teacher-student relationship. Additionally, compared to male teachers, female teachers have a significantly greater impact on TSR under high care conditions, possibly because female teachers pay more attention to emotions and care. They are more meticulous in observing students’ emotional states, paying attention to students’ needs, and providing support and help whenever possible (Card et al., 2022). In this context, female teachers tend to use emotionally rich communication methods and expressions of care, which helps them establish a closer emotional connection with students.

Considering that the data from the CEPS used in Study 1 is cross-sectional, it can only reveal the association between teacher care and TSR, without determining the causal relationship. To better elucidate the causal relationship between teacher care and TSR, Study 2 builds upon the foundational data from Study 1 and employs dynamic tracking survey data from the second phase of the 2014–2015 academic year of CEPS, as well as more rigorous causal inference methods such as the cross-lagged model, making the research conclusions more robust.

Study 2 descriptive statistical results indicate a slight decline in teacher care from seventh to eighth grade, with a concurrent downward trend in TSR. Burnout Theory offers insights into the potential reasons for this phenomenon. As seventh grade marks a new phase in students’ academic lives, teachers typically invest considerable time and energy to meet students’ needs and help them adapt to new learning environments and challenges (Madigan and Kim, 2021). However, over time, the prolonged demands of educational work may lead to emotional exhaustion among teachers, which could reduce their care and positive interactions with students, thereby diminishing the quality of TSR (Wang Y. et al., 2024). There is a significant positive correlation between pre-test and post-test measures of teacher care and TSR. These findings are consistent with previous research, suggesting an intrinsic connection between the two (Johnston et al., 2022).

Based on the cross-lagged model, this study further validates the bidirectional predictive relationship between teacher care and TSR, highlighting the significant role of teacher care and TSR in promoting the learning and overall educational quality of Chinese middle school students. Specifically, first, teacher care significantly and positively predicts TSR, which is consistent with previous research. When teachers care about their students, they create a safe and confident atmosphere, thereby enhancing the positive relationship between teachers and students as perceived by parents (Tripon, 2021). A teacher’s concern can enhance students’ willingness to interact openly with teachers, disclose emotions, and share personal information, thereby fostering the relationship with the teacher (Lavy and Naama-Ghanayim, 2020). Second, TSR can significantly positively predict teacher care, indicating that the quality of TSR in the first year can predict the level of teacher care in the second year. Interpersonal Interaction Theory emphasizes the importance of emotions and interactions in interpersonal relationships (Roche and Cain, 2021). Good relationships established between teachers and students can promote more effective communication and understanding, allowing teachers to better grasp students’ needs and emotions, triggering teacher care behaviors, and establishing a caring relationship with students.

The study did not find gender differences in the cross-lagged model, which may be due to biases in the selected sample, with the sample size of male teachers being lower than that of female teachers, affecting the significance of gender differences. In summary, these data indicate that teacher care and TSR not only exhibit stability over time but also have a reciprocally influential relationship.

## 6 The practical implications and future recommendations

In terms of practical significance, by exploring the impact of parental perceived teacher care on TSR, this study provides an empirical foundation for educators to facilitate the development and implementation of more humanized and effective educational strategies. It not only helps us better understand the role of parents and teachers in the growth process of students but also offers guidance for educational practice. This guidance assists parents and teachers in better understanding and meeting the needs of students in their daily work, thereby establishing more harmonious TSR and creating favorable conditions for the holistic development of students.

As generations of students become more and more different, teachers must consider the ways of getting along with their students (Tripon, 2022). On one hand, while completing their daily teaching tasks and providing academic support to students, teachers should also offer emotional support and care, making students feel respected, understood, and accepted, thereby imparting a sense of warmth and concern (Wang Y. et al., 2024). For instance, a simple greeting such as “How have you been?” or showing further attention and care when a student appears uneasy can provide comfort and support to students. On the other hand, teachers can enhance their emotional bonds with students through conversations, walks, and various recreational activities, gaining a deeper understanding of students’ interests, aspirations, and academic situations. By caring with students and considering their perspectives, teachers can establish friendly TSR (Guzzardo et al., 2021).

The present study has several limitations that warrant attention. Firstly, due to the constraints of the existing dataset, the information on teacher care and TSR was derived exclusively from parent questionnaires and it is possible that the parents would have noticed something of the answers of the others (some of them were surveyed in groups). Parental perceptions may not accurately reflect the actual dynamics between students and teachers. Future research should strive to optimize the survey methods and combining multiple sources of information, such as in-depth interviews with students and their parents, as well as direct observations (Deterding and Waters, 2021), to assess the perceptions of students, teachers, and parents regarding teacher care and TSR, thereby gaining a more comprehensive and accurate understanding. Secondly, there is an imbalance in the sample sizes of male and female teachers in this study, which may affect the accurate assessment of gender differences. Future studies should explore whether there are moderating effects of teacher gender on teacher care and TSR under conditions where the sample sizes of male and female teachers are equal or similar. Thirdly, the data used in this study is relatively old, and the time span is short, with data collected only at two time points. This may be insufficient to reveal the long-term changes in parental perceived teacher care or its sustained impact on TSR. Future research could consider updating the database and extending the time span of longitudinal studies, covering at least 3 to 5 years, or even longer. This would allow for a clearer observation of the dynamic changes between parental perceived teacher care and TSR, as well as any potential long-term effects. Lastly, the current study does not take into account the impact of the COVID-19 pandemic on parental perceived teacher care and TSR. Future research should consider the influence of major social events, such as the COVID-19 pandemic, on the educational environment and parents’ perceptions (Zaccoletti et al., 2020). A comparison of pre- and post-pandemic data could be conducted to explore how social changes have affected TSR, particularly in special contexts such as online education and changes in educational policies.

## 7 Conclusion

This study utilizes the nationally representative CEPS data to conduct an empirical analysis of the impact of parental perceived teacher care on TSR for the first time. Study 1 found that there was a significant positive correlation between teacher gender, parental perceived teacher care and TSR. The analysis of simple regulation effect found that teacher gender plays a regulating role between parental perceived teacher care and TSR. Further simple slope analysis found that the effect of parent perceptions of female teacher care on the TSR was significantly greater than that of male teachers. Study 2 results reveal a slight decline in parental perceived teacher care from seventh to eighth grade, with a concurrent downward trend in TSR. This decline necessitates that teachers and parents pay attention to the changes in the process of students’ grade promotion, and strengthen communication and cooperation to promote the effective transmission of teacher care and the positive development of teacher-student relationship. Additionally, at both time points, there is a significant positive correlation between teacher care and TSR. Further analysis through the cross-lagged model reveals a bidirectional predictive relationship between teacher care and TSR. This underscores the crucial role of both perceptions of teacher care and TSR in the growth process of middle school students. When parents perceive that their child’s teacher is caring and supportive, it can reinforce the positive effects of teacher care on TSR. This perception can enhance the trust and communication between parents, teachers, and students, creating a more supportive educational environment that fosters better TSR and overall student development.

## Data Availability

The raw data supporting the conclusions of this article will be made available by the authors, without undue reservation.
