# Supplementary material for: The impact of teacher care on teacher-student relationship: evidence from cross-sectional and longitudinal data
Source: Front Psychol. 2025 Jan 28;16:1551081. doi: 10.3389/fpsyg.2025.1551081 (PMC11810937; doi:10.3389/fpsyg.2025.1551081)
Supplement: Supplementary file 1 [file Table_1.DOCX]

Supplementary Material

# Supplementary Tables

Table 1. Descriptive Statistics and Correlation Analysis

| Variable | *M* | *SD* | 1 | 2 | 3 |
| --- | --- | --- | --- | --- | --- |
| Teacher Gender | 0.63 | 0.48 | 1 |  |  |
| Teacher Care | 4.26 | 0.74 | 0.14^***^ | 1 |  |
| TSR | 3.32 | 0.59 | 0.13^***^ | 0.57^***^ | 1 |

Note: ^*^*p* < 0.05, ^**^*p* < 0.01, ^***^*p* < 0.001.

Table 2. Simple Moderation Effect Analysis

| Variable | | Fitting Index | | | Standardized Coefficients | | |
| --- | --- | --- | --- | --- | --- | --- | --- |
| Result Variable | Prediction Variable | *R* | *R²* | *F* | *β* | *t* | 95%CI |
| TSR | Teacher Care | 0.58 | 0.33 | 1461.97 | 0.55^***^ | 86.99 | [0.54,0.56] |
|  | Teacher Gender |  |  |  | 0.04^***^ | 6.89 | [0.03,0.06] |
|  | Teacher Care×Teacher Gender |  |  |  | 0.02^***^ | 3.70 | [0.01,0.03] |

Note: ^*^*p* < 0.05, ^**^*p* < 0.01, ^***^*p* < 0.001.

Table 3. Descriptive Statistics of Teacher Care and TSR

| Year | Variables | N | Mean | Standard Deviation | Min | Max | Skewness | Kurtosis |
| --- | --- | --- | --- | --- | --- | --- | --- | --- |
| Year1 | Teacher Care | 5393 | 4.39 | 0.68 | 1 | 5 | -1.079 | 1.149 |
|  | TSR | 5393 | 3.46 | 0.54 | 1 | 4 | -.695 | .457 |
| Year2 | Teacher Care | 5393 | 4.34 | 0.74 | 1 | 5 | -1.207 | 1.690 |
|  | TSR | 5393 | 3.27 | 0.58 | 1 | 4 | -.494 | .401 |

Table 4. Correlation Analysis of Teacher Care and TSR

| Year | Variables | 1 | 2 | 3 | 4 |
| --- | --- | --- | --- | --- | --- |
| Year1 | 1.Teacher Care | 1 |  |  |  |
|  | 2.TSR | 0.54^***^ | 1 |  |  |
| Year2 | 3.Teacher Care | 0.13^***^ | 0.13^***^ | 1 |  |
|  | 4.TSR | 0.12^***^ | 0.12^***^ | 0.56^***^ | 1 |

Note: ^*^*p* < 0.05, ^**^*p* < 0.01, ^***^*p* < 0.001.

## 2 Supplementary Figures


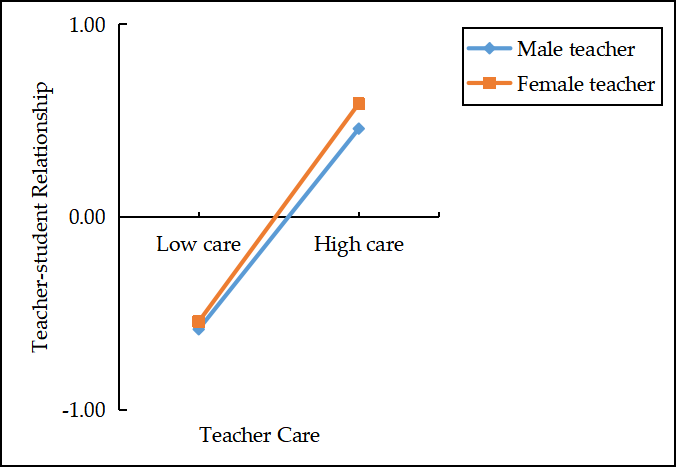


**Supplementary Figure 1.** Moderation Effect of teacher gender on teacher care and TSR.


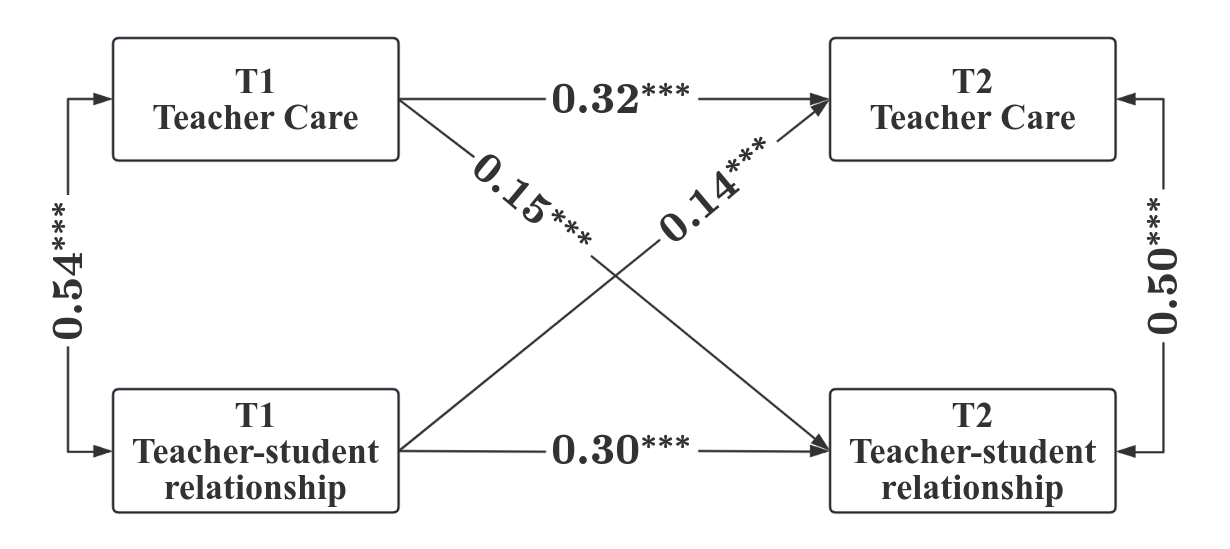


**Supplementary Figure 2.** Bidirectional relationships between teacher care and TSR. Note: We use “^*^” to indicate a statistically significant relationship (*p* < 0.05) between variables.
